# Spatiotemporal Alterations in Gait in Humanized Transgenic Sickle Mice

**DOI:** 10.3389/fimmu.2020.561947

**Published:** 2020-10-15

**Authors:** Stacy Kiven, Ying Wang, Anupam Aich, Donovan A. Argueta, Jianxun Lei, Varun Sagi, Madhushan Tennakoon, Saad J. Bedros, Nils Lambrecht, Kalpna Gupta

**Affiliations:** ^1^Hematology/Oncology, Department of Medicine, University of California, Irvine, Irvine, CA, United States; ^2^Division of Hematology, Oncology and Transplantation, Department of Medicine, Vascular Biology Center, University of Minnesota, Minneapolis, MN, United States; ^3^Department of Anesthesia, Stark Neuroscience Research Institute, Indiana University School of Medicine, Indianapolis, IN, United States; ^4^College of Science & Engineering, University of Minnesota, Minneapolis, MN, United States; ^5^Pathology and Laboratory Medicine, Long Beach VA Healthcare System, Long Beach, CA, United States; ^6^Southern California Institute for Research and Education, Long Beach VA Healthcare System, Long Beach, CA, United States

**Keywords:** purkinje cell, sickle cell disease, gait, hyperalgesia, histopathology

## Abstract

Sickle cell disease (SCD) is a hemoglobinopathy affecting multiple organs and featuring acute and chronic pain. Purkinje cell damage and hyperalgesia have been demonstrated in transgenic sickle mice. Purkinje cells are associated with movement and neural function which may influence pain. We hypothesized that Purkinje cell damage and/or chronic pain burden provoke compensatory gait changes in sickle mice. We found that Purkinje cells undergoe increased apoptosis as shown by caspase-3 activation. Using an automated gait measurement system, MouseWalker, we characterized spatiotemporal gait characteristics of humanized transgenic BERK sickle mice in comparison to control mice. Sickle mice showed alteration in stance instability and dynamic gait parameters (walking speed, stance duration, swing duration and specific swing indices). Differences in stance instability may reflect motor dysfunction due to damaged Purkinje cells. Alterations in diagonal and all stance indices indicative of hesitation during walking may originate from motor dysfunction and/or arise from fear and/or anticipation of movement-evoked pain. We also demonstrate that stance duration, diagonal swing indices and all stance indices correlate with both mechanical and deep tissue hyperalgesia, while stance instability correlates with only deep tissue hyperalgesia. Therefore, objective analysis of gait in SCD may provide insights into neurological impairment and pain states.

## Introduction

Chronic pain and organ damage are major comorbidities of sickle cell disease (SCD) (1–5). Organ pathology has been observed in humanized sickle mice, which show several clinical features of SCD including chronic pain and multi-organ pathology (6, 7). Interestingly, Purkinje cell damage in the brain of the HbSS-BERK mice has been previously observed (6). Purkinje cells are the principal output neurons of the cerebellar cortical microcircuit, and thus play a fundamental role in coordinating cerebellar function by integrating massive excitatory synaptic input, as well as firing high–frequency and highly regular action potentials in the absence of synaptic drive (8). Purkinje cell malfunction has been observed in mouse models of several forms of ataxia, which exhibit alterations in gait (9, 10). Since Purkinje cells regulate the sensory-motor functions, it is likely that the pathological changes in Purkinje cells of sickle mice may contribute to alterations in gait.

The Pain in Sickle Cell Epidemiology Study (PiSCES) cohort demonstrated that the adult sickle population suffers from knee-skin, lower back, and hip pain for about one-third of the chronic pain days (11). Additionally, bone complications-associated joint pain is common in SCD with about 50% adults developing avascular osteonecrosis due to loss of blood supply to the femoral head in addition to osteoporosis and osteopenia (12–14). Femurs from transgenic sickle mice exhibit altered microstructure with 40% reduced mechanical strength compared to control mice (15). In addition to sudden and intractable acute pain, chronic joint and back pain in combination with reduced bone strength could contribute to difficulty in mobility and subsequent postural adjustment to compensate for the pain, i.e., change in gait (16). Therefore, gait patterns in SCD can be a result of compensatory adaptation to avoid movement-evoked pain in addition to alterations in gait due to Purkinje cell damage.

Analysis of dynamic gait parameters such as walking speed, stand/swing duration, and step length are of interest for assessing locomotion function in many motion-affected human conditions (17). Automated gait measurement has also been used as an objective measure of pain in inflammatory and neuropathic pain models in mice (18). In the present study, we evaluated the Purkinje cell pathology and gait changes in transgenic HbSS-BERK sickle mice compared to HbAA-BERK control mice. We utilized the novel video-based automated “MouseWalker” system to analyze gait and simultaneously examine the correlation of hyperalgesia with gait in sickle mice.

## Materials and Methods

### Animals

We used a total of 24 mice consisting of control (HbAA-BERK) and sickle (HbSS-BERK), hereafter referred to as control and sickle mice, respectively. All mice were bred and raised in-house with *ad libitum* access to food and water on a 12-h light/dark cycle in conventional housing and used at ~3.5 months of age (7). Control and sickle mice are homozygous for knockout of both murine α and β globins. Control mice express normal human hemoglobin A and sickle mice express human α and β^S^ globin chains with >99% human hemoglobin S, but no murine α or β globin (19). Sickle mice show similarities with human SCD including erythrocytic sickling, intravascular hemolysis, reticulocytosis, severe anemia (hematocrit, 10–30%), leukocytosis, elevation of inflammatory cytokines, pulmonary congestion, and shortened life-span (6, 20, 21). Interestingly, increased multiorgan infarcts and pyknotic Purkinje cells have been observed in sickle mice compared to non-sickle control mice (6, 22). Sickle pain is characteristically complex in nature with nociceptive, neuropathic, and inflammatory components in its etiology (1, 2). Humanized HbSS-BERK sickle mice exhibit enhanced mechanical, thermal and deep tissue hyperalgesia compared to control mice (23, 24). All mice were validated by phenotyping for sickle and normal human hemoglobin by isoelectric focusing as previously described (7).

### Assessment of Hyperalgesia

Mice were acclimatized to each test protocol in a quiet room at constant temperature, and tested for mechanical (von Frey) and spontaneous musculoskeletal/deep (grip force) as previously described (7, 23). Behavioral tests were performed consecutively at a 5-min interval between tests, in a double-blind manner.

Mechanical hyperalgesia was measured by applying a 1.0 g (4.08 mN) von Frey (Semmes-Weinstein) monofilament (Stoelting Co, Wood Dale, IL) to the mid-plantar surface of each hindpaw for a total of 10 trials per hindpaw with a 5-s inter-stimulus interval, and paw withdrawal frequency (PWF) was recorded. Deep tissue/musculoskeletal hyperalgesia was assessed by placing mice on a wire-mesh gauge by their forepaws, and the peak grip force exerted in grams was recorded by a computerized grip-force meter (SA Maier Co, Milwaukee, WI).

### Histopathological Analysis

Whole brains were collected immediately following euthanasia and fixed in 10% formalin (575A-14 43 mm; Medical Chemical Corporation, Torrance, CA, USA). Using routine histology methods, fixed tissues were processed, embedded in paraffin, and sections were stained with hematoxylin and eosin (H&E) or with routine immunohistochemical methods. H&E stained sections were analyzed using a Nikon eclipse 50i light microscope equipped with a 10 × ocular with an edged-in 1 mm scale with 0.01 mm divisions calibrated to the 10 × objective. Images were acquired by an attached Nikon DS-Fi1 camera.

### Immunohistochemistry

Slides with 5 μm sections of brain were deparaffinized and rehydrated with Histo-Clear (National Diagnostics, Atlanta, GA, USA) and ethanol gradient, respectively. Sections were processed for cleaved caspase-3 (an activated form) detection to assess Purkinje cell apoptosis. Antigen retrieval was performed with Target Retrieval Solution (Agilent, Santa Clara, CA, USA), and subsequently processed with ABC detection kit (ab64261; Abcam, Cambridge, MA, USA). Endogenous peroxidase was inactivated by incubating with manufacturer-provided hydrogen peroxide solution (Abcam) for 10 min. Non-specific binding was blocked with blocking solution (Abcam) for additional 10 min. Sections were incubated for 1 h at room temperature in 1:500 rabbit anti-mouse cleaved caspase-3 primary antibody (ab2302; Abcam), then in prediluted biotinylated goat-anti rabbit secondary antibody (Abcam). Slides were then incubated for 10 min with streptavidin peroxidase and stained with DAB chromagen (Abcam), after which samples were counterstained with hematoxylin. Slides were dehydrated with ethanol gradient and Histo-Clear before cover slipping. Stained sections were examined and analyzed by a board-certified surgical pathologist with expertise in mouse histopathology, in a double-blind manner.

### Assessment of Gait

The MouseWalker system was assembled in our laboratory following the design described for gait measurement of mice (25). Each mouse was individually placed in a transparent corridor (8 × 80 cm^2^) with acrylic glass floor panel mounted with LED lights, which produced a detectable touch sensor. Total internal reflection (TIR) of the LEDs in the acrylic surface was measured with embedded light sensors. During mouse natural walking gait, foot contact disrupted TIR causing frustrated total internal reflection (fTIR) within the transparent material. The fTIR-illuminated points of contact were detected by a high-speed CMOS camera (Lumenera Lt425C, Lumenera Corporation, Ottawa, Ontario) with a 16 mm lens. Constant background lighting was established with a backlit board placed 40 cm over the corridor, which comprised two colored LEDs and aluminum bar sets (HL-LS5050_RGB300NW44K, HitLights, LA, USA; 9001 K25, MacMaster-Carr, IL, USA). The light color was set by a controller box and remote control. The background light and fTIR light were set to blue (intensity: 60%) and white (intensity: 100%), respectively, for optimum video quality. High-performance digital video recording software was used (StreamPix 7, Norpix, Montreal, Quebec) at a resolution of 2048 × 2048 for data analysis. Mice were habituated in the corridor during the 3 days prior to experiment date. Four videos were acquired of each mouse and 2 videos of uninterrupted walking were selected and analyzed. The MouseWalker program was run in Matlab (The Mathworks, MA, USA). Both the program and manual are available online (biooptics.markalab.org). Matlab software was used to distinguish the footprints from background, and convert the videos to grayscale prior to analysis in the MouseWalker software. The mislabeled footprints or body features were manually adjusted following automatic detection with MouseWalker software. Gait-related parameters including walking speed, stance instability, and stance duration were extracted and exported from the MouseWalker software to determine correlation with measures of hyperalgesia.

### Statistical Analysis

All data were analyzed using Prism software (v 6.0f, GraphPad Prism Inc., San Diego, CA). Data between groups were analyzed using 1-way analysis of variance (ANOVA) with *post hoc* Bonferroni's multiple comparisons. Data within groups were compared using 2-way repeated measures ANOVA with *post hoc* Bonferroni's/Sidak's multiple comparisons tests. Gait parameters were analyzed using Student's unpaired two-tailed *t*-test, and Pearson correlation analysis was performed to detect associations of gait parameters with hyperalgesia—normality of data for correlation analysis was determined with Anderson-Darling test. A *p* < 0.05 was considered statistically significant. All data are represented as mean ± SEM.

## Results

### Increased Purkinje Cell Apoptosis in the Cerebellum of Sickle Mice

**Figure 1:**
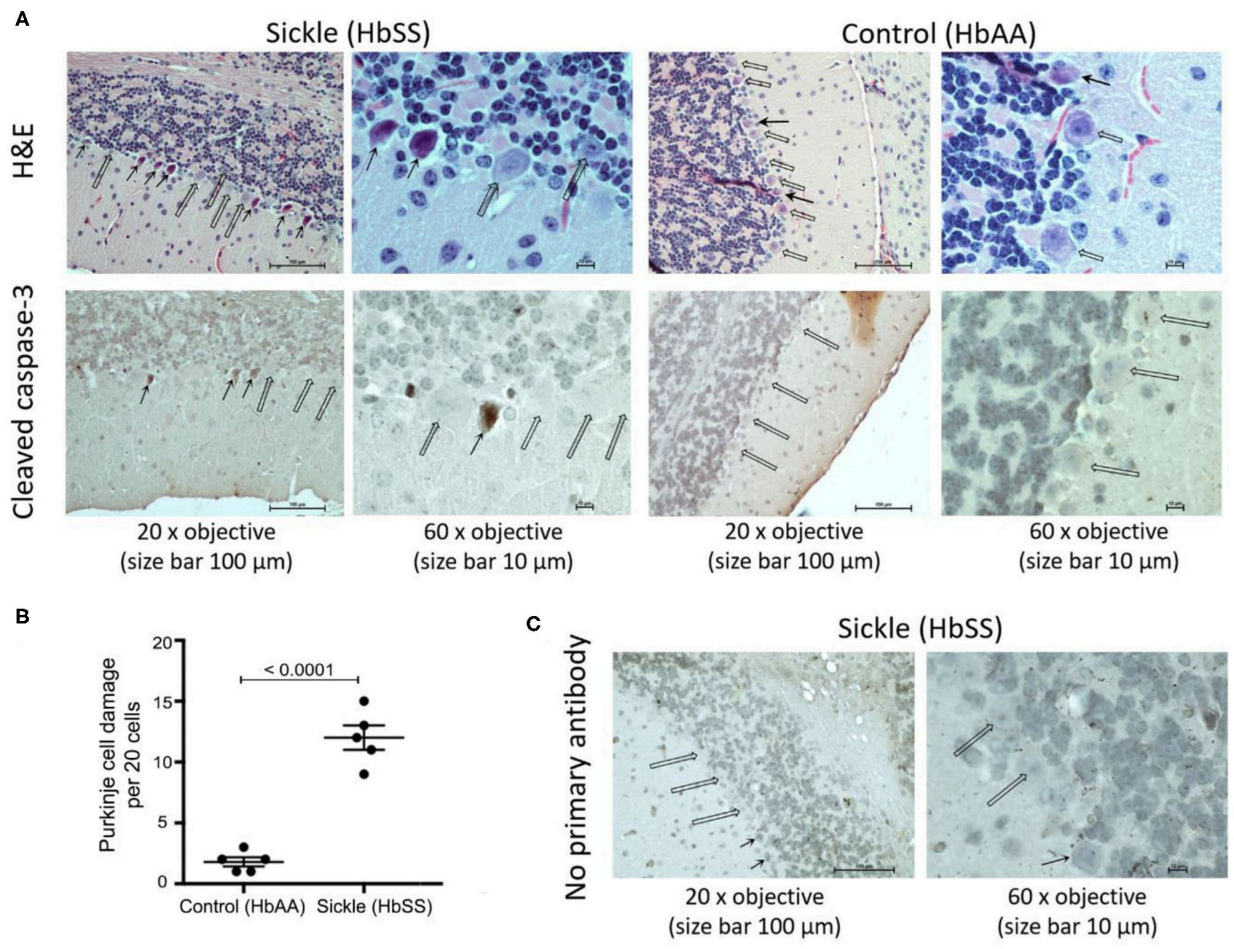
Sickle mice show increased Purkinje cell damage compared to control mice. Cerebellum from ~3.5 month old female HbSS-BERK sickle and HbAA-BERK control mice. **(A)** H&E and capase-3 stained sections of cerebellum at 20x objective, size bar 100 μm and at 60x objective, size bar 10 μm. Open arrows: normal Purkinje cells with well-organized nucleus. Solid arrows: apoptotic Purkinje cells with smudged and irregular nuclei with condensed chromatin. **(B)** Quantification of Purkinje cell apoptosis per 20 cells/mouse brain from 5 control 5 sickle mice (unpaired two-tailed *t*-test). **(C)** Incubation of sickle mice brain sections without primary antibody showed no cross reactivity with secondary antibody as a negative control. Open arrows: normal Purkinje cells. Solid arrows: apoptotic Purkinje cells with smudged irregular nuclei.

**Figure 2:**
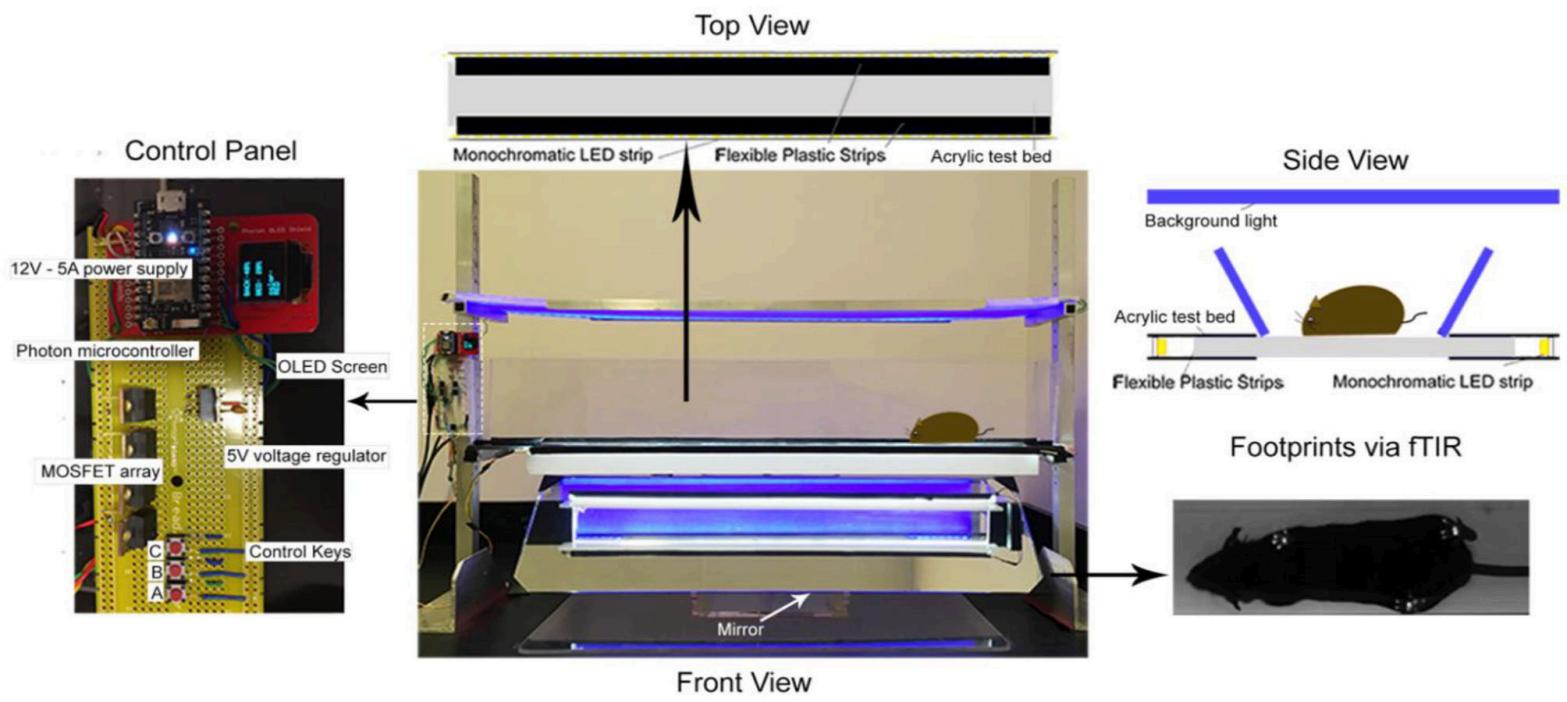
MouseWalker Apparatus. Each mouse was individually placed in a transparent corridor (8 × 80 cm^2^) with acrylic glass floor panel mounted with LED lights, which produced a detectable touch sensor. Total internal reflection (TIR) of the LEDs in the acrylic surface was measured with embedded light sensors. During natural walking, foot contact disrupted TIR causing frustrated total internal reflection (fTIR) within the transparent material. The fTIR illuminated points of contact, which were detected by a high-speed CMOS camera (Lumenera Lt425C, Lumenera Corporation, Ottawa, Ontario) with a 16 mm lens. Constant background lighting was established with a backlit board placed 40 cm over the corridor, which comprised two-colored LEDs and aluminum bar sets (HL-LS5050_RGB300NW44K, HitLights, LA, USA; 9001 K25, MacMaster-Carr, IL, USA). The light color was set by a controller box and remote control. The background light and FTIR light were set to blue (intensity: 60%) and white (intensity: 100%), respectively, for optimum video quality. High-performance digital video recording software was used (StreamPix 7, Norpix, Montreal, Quebec) at a resolution of 2048 × 2048 for data assessment. Mice were habituated in the corridor during 3 days before experiment date. Four videos were taken of each mouse and 2 videos of uninterrupted walking were selected and analyzed. The MouseWalker program was developed and compiled in MATLAB (The Mathworks, MA, USA). Matlab software was used to distinguish the footprints from background and convert the videos to grayscale prior to analysis in the MouseWalker software. The mislabeled footprints or body features were manually adjusted following automatic detection with MouseWalker software. Gait-related parameters such as stance instability, walking speed, and swing/stance duration were extracted and exported from the MouseWalker software to determine correlation with measures of hyperalgesia.

H&E staining shows that Purkinje cells in control mice have a well-shaped nucleus, finely granular distributed chromatin, and an intact nucleolus (Figure 1A, open arrows). In contrast, H&E stained sections from sickle mice brains show morphological features of apoptosis in Purkinje cells with a smudged nucleus with condensed chromatin and lack of a well-formed nucleolus (Figure 1A, solid arrows). Cellular apoptosis was further validated by immunostaining the brain section with cleaved caspase-3 (active form), a critical protease in the pro-apoptosis pathway (26–29). Purkinje cells of control mice did not show the expression of cleaved caspase-3, consistent with histopathological observations of intact nucleus and well-shaped cellular morphology. However, Purkinje cells of sickle mice clearly showed cleaved caspase-3 immunoreactivity, consistent with histopathology suggestive of apoptosis (Figure 1A, solid arrows). Purkinje cell apoptosis was quantified by counting the number of damaged cells per 20 cells/brain of each mouse. Sickle mice showed significantly more Purkinje cell apoptosis compared to control mice (Figure 1B; *p* ≤ 0.0001). These results (along with previous observations) indicate that Purkinje cell apoptosis is a characteristic feature of sickle pathobiology (Figure 1C). As Purkinje cell apoptosis is often associated with motor dysfunction, we tested whether sickle mice with chronic pain display altered gait compared to control mice, using our custom-built in-house *MouseWalker* platform (Figure 2).

### Spatiotemporal Changes in Gait in Sickle Mice

#### Hyperalgesia and Stance Instability Are Significantly Higher in Sickle Mice

**Figure 3:**
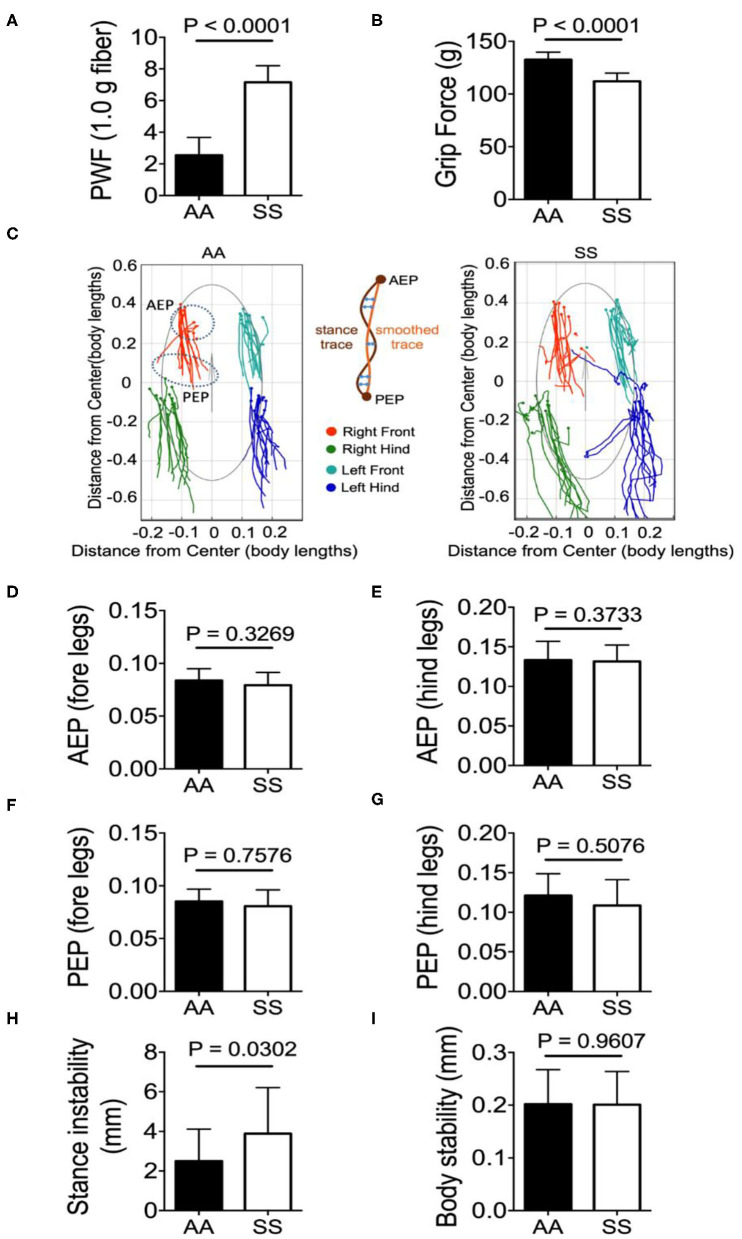
Sickle mice with chronic pain present stance instability. Hyperalagesia and gait parameters were measured in the test groups of untreated HbSS-BERK sickle (SS) and HbAA-BERK control (AA) mice. **(A)** Mechanical hyperalgesia assessed by Paw Withdrawal Frequency (PWF) in response to 1.0-g von Frey filaments. Mechanical and **(B)** deep tissue hyperalgesia assessed by a computerized grip-force measure. **(C)** shows representative stance trace of one sickle (right) and control mouse (left) walking at 21.59 and 21.20 cm/s, respectively. The traces were determined by measuring the position of the stance phase footprints relative to the body center. The anterior extreme position (AEP) indicates stance onset and the posterior extreme position (PEP) indicates stance offset (positions encircled by a dashed line for the right fore paw). For each stance trace (maroon), a smoothed trace is generated (using data from every five frames; yellow trace), and the average of the difference between these two lines (orange arrows) corresponds to the stance instability. **(D,E)** Perpendicular AEP and **(F,G)** perpendicular PEP plots for both fore and hind legs, respectively. **(H,I)** show stance instability and body instability, respectively. Gait parameters were analyzed using Student's unpaired two-tailed *t*-test. Significance was determined by *t*-test (unpaired, two-tailed). A *p* < 0.05 was considered statistically significant. All data are represented as mean ± SEM. [*n* = 7 per group in **(A,B,D–I)**].

Mechanical hyperalgesia was assessed by quantifying the PWF in response to 10 applications of von Frey monofilaments to the hind paw of mice. Higher PWF is indicative of more pain, which was the case in sickle mice compared to control mice. Deep tissue hyperalgesia was measured by assessing the grip force applied by the fore limbs of the mouse while pulling a wire gauge. Mice with more pain exert lower force and sickle mice showed lower grip force compared to control mice. Together, these data show that sickle mice demonstrate significantly increased mechanical (Figure 3A; *p* < 0.0001) and deep tissue/musculoskeletal hyperalgesia (Figure 3B; *p* < 0.0001) compared to control mice.

All the gait and body parameters extracted from *MouseWalker* software were compared between sickle and control mice to examine the difference in walking speeds within the range of 10–40 cm/s. A single gait cycle of movement is known as stride that is divided into two major phases: stance phase and swing phase. The phase/duration in which the paw/foot stays in touch with the ground is referred to as stance phase and conversely, in the swing phase the paw/foot is not in contact with the ground. A major gait measure is the stance trace during the stride that is defined as the position of the foot relative to the center of the body from paw touchdown (anterior extreme position, AEP) to the end of the stance phase (posterior extreme position, PEP) and reflects the amount of body wobble during stance phases (25). Representative stance traces from a sickle and control mouse each with similar walking velocity (control: 14.50 cm/s, sickle: 14.56 cm/s) are presented (Figure 3C). Compared to the control mice, the stance traces of sickle mice at ~14.50 cm/s displayed large variations of AEP and PEP for both fore- and hind-limbs with fore-limb traces being less variable than the hind-limb traces, although no statistical difference was observed (Figures 3D–G; *p* = 0.3269, *p* = 0.3733, *p* = 0.7576, *p* = 0.5076, respectively). Interestingly, sickle mice with pain showed significantly higher stance instability compared to control mice (Figure 3H; *P* = 0.0302) while the body stability remained similar among the two groups (Figure 3I; *p* = 0.9607). The stance instability is determined from the stance linearity index, which reflects the linearity of the stance traces by calculating the average difference between the actual stance trace and a smoothed version of the trace (25). Thus, higher stance instability in sickle mice may be indicative of locomotion abnormality either due to hyperalgesia or due to motor dysfunction suggested by Purkinje cell damage.

#### Stance and Swing Phase Durations Are Significantly Longer and Posit Non-uniform Distribution for Sickle Mice

**Figure 4:**
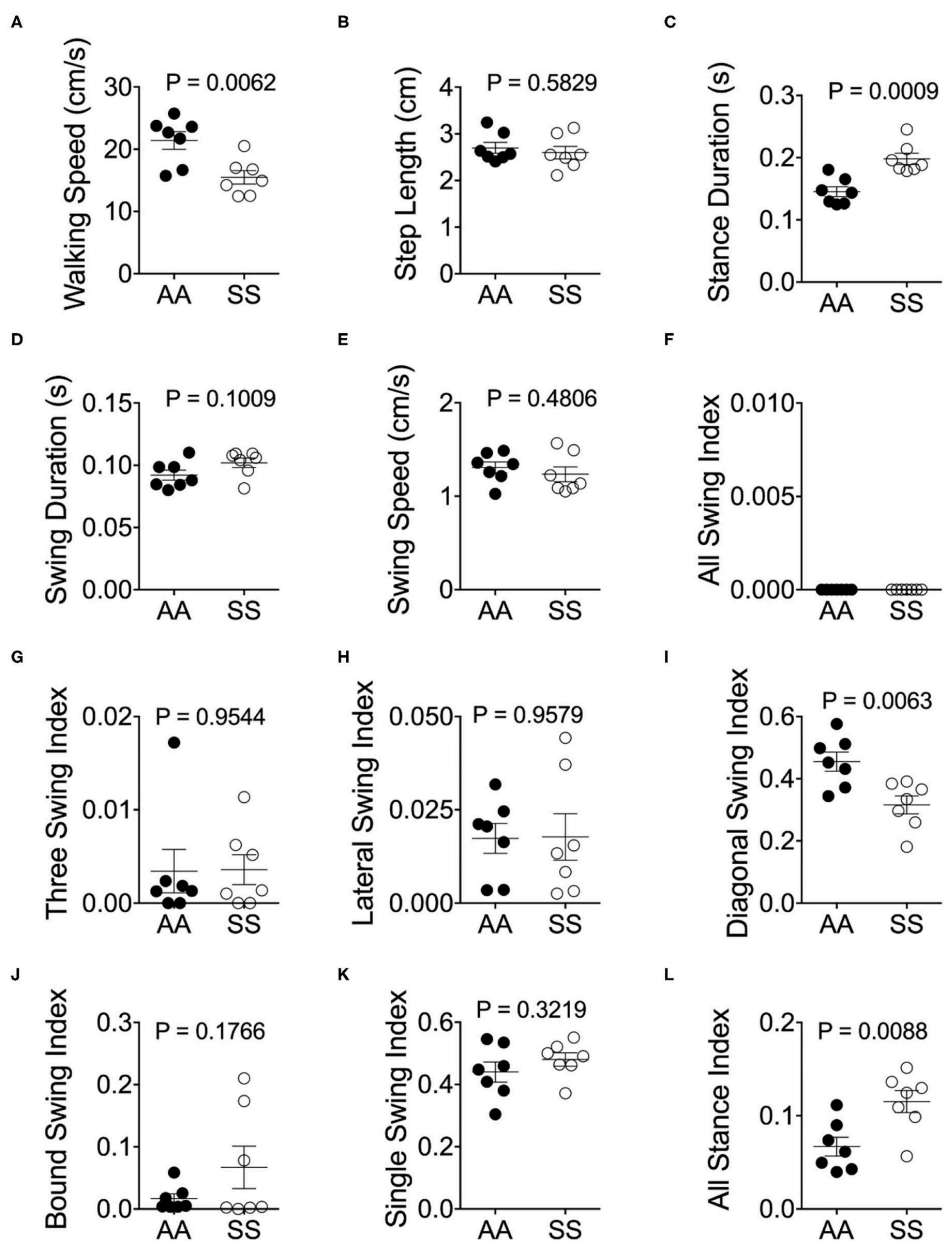
Alterations of gait parameters in sickle mice. Gait parameters were analyzed and compared between HbSS-BERK sickle (SS) and HbAA-BERK control (AA) mice The average walking speed **(A)**, step length **(B)**, stance **(C)**, swing duration **(D)**, and swing speed **(E)**, were compared at walking speed between 10–40 cm/s. The seven stance indices of all-leg swing analyzed were **(F)**, three-leg swing **(G)**, lateral swing **(H)**, diagonal swing **(I)**, bound swing **(J)**, single swing **(K)**, and all stance **(L)**. Gait parameters were analyzed using Student's unpaired two-tailed *t*-test. Significance was determined by *t*-test (unpaired, two-tailed). [*n* = 7 per group in **(A–E)**]. **(F–H)** Individual results of 3 videos per mouse. A *p* < 0.05 was considered statistically significant. All data are represented as mean ± SEM.

Mendes et al. demonstrated that the step distances of C57BL/6J mice were exponentially increased with faster velocities (25). However, we have observed that the majority of sickle mice walked at a visibly lower velocity in comparison with control mice (Figure 4A; *p* = 0.0062), although the step lengths were similar (Figure 4B; *p* = 0.5829). Consistent with Mendes' study (25), we also observed a large variation in the stance duration (Figure 4C; *p* = 0.0009) and smaller variation of swing duration (Figure 4D; *p* = 0.1009) in both sickle and control mice which exponentially decreased as the speed increased [data not shown]. Lastly, stance phases lasted longer than swing phases at all speeds in both mice (Figures 4C,D). These data indicate that sickle mice inherently suffer from altered walking gait patterns that may have resulted from hyperalgesia/motor dysfunction.

#### Diagonal Swing Indices Are Decreased and All Stance Indices Are Significantly Increased for Sickle Mice

**Figure 5:**
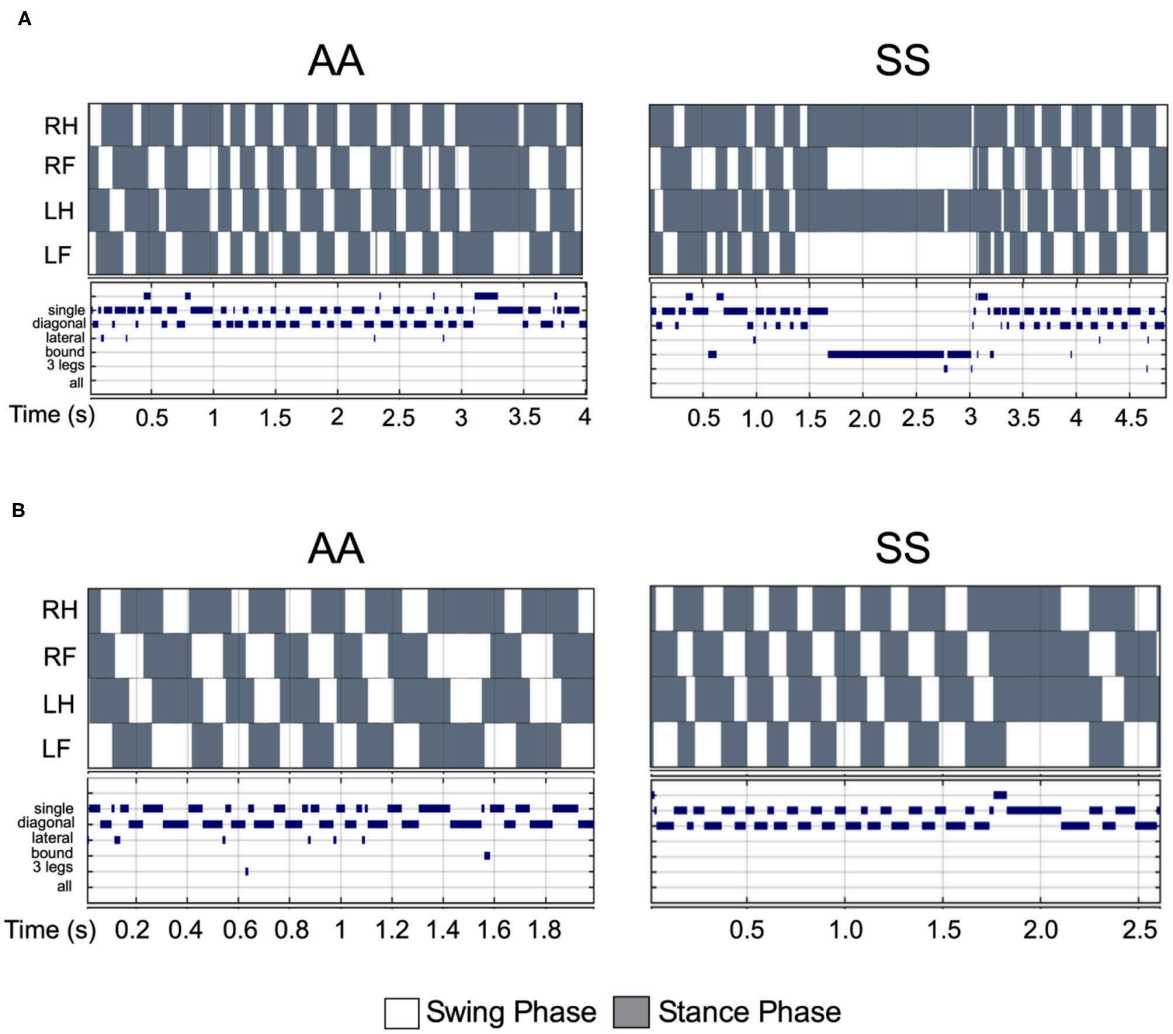
Comparison of step pattern and modalities between sickle and control mice under same walking velocity. **(A)** Representative stance trace and gait parameters of inter-leg coordination of HbSS-BERK sickle (SS) and HbAA-BERK control (AA) mice with walking speeds at 14.50 and 14.56 cm/s, respectively. **(B)** Representative stance trace and gait parameters of inter-leg coordination of SS and AA mice with walking speeds at 21.2 and 21.59 cm/s, respectively. The upper images of all graphs are gait patterns are composed of two phases which are swing phase (white areas) and stance phase (gray areas). The lower panel shows step combinations.

Walking/running gait involves swing modalities depending on how the legs are being moved/lifted during swing phase. We analyzed seven walking modes of leg combinations in sickle and control mice: no swing (stance), single-limb swing (lifting of one limb, four modes), diagonal-limb swing (lifting left/right fore limb with right/left hind limb, two modes), lateral-limb swing (both left or both right limbs, two modes), bound-limb swing (both hind or both fore legs, two modes), three-limb swing (lifting of any three legs, 4 modes), or all-limb swing (lifting of all limbs, one mode) (Figure 4E). A walking speed of 52.8 cm/s is the transitional speed from walking to running in which situation swing duration basically surpasses stance duration (25). Since we have only included sickle and control mice with walking speeds (10–40 cm/s) instead of running (>52.8 cm/s) speed, all swing indices turned out to be zero for both control and sickle mice, meaning at no point of time all the limbs were in the air (Figure 4F). Three limb swing and lateral swing indices approach “0” in both groups (Figures 4G,H; *p* = 0.9544 and *p* = 0.9579, respectively). Diagonal swing conformations were the most representative configuration and constituted more than 50% of the frames in our control mice and significantly reduced to 40% in sickle mice (Figure 4I; *p* = 0.0063). Moreover, BERK sickle mice showed increased but insignificant bound (Figure 4J; *p* = 0.1766) and single swing (Figure 4K; *p* = 0.3219) modality (vs. control mice). Consistent with our observation during the whole course of the experiment, the frequent hesitant stops in sickle mice resulted in significantly increased all stance index (Figure 4L; *p* = 0.0088). Moreover, compared to control mice, sickle mice with nearly the same walking speed showed unequally distributed gait patterns with more frequent swing phases (increased appearance of white squares) and extended stance duration (elongated gray square) at certain points, leading to an incoherent walking speed with frequently altered gait patterns (Figures 5A,B). Cumulatively, these data indicate an important behavioral aspect of sickle mice—a hesitation in breaking inertia to move on to the next phase, be it swing or stance phase. This hesitation may be a reflection of motor dysfunction due to Purkinje cell damage or may result from fear or anticipation of movement-evoked pain.

### Selective Spatial and Temporal Gait Parameters Correlate With Hyperalgesia

**Figure 6:**
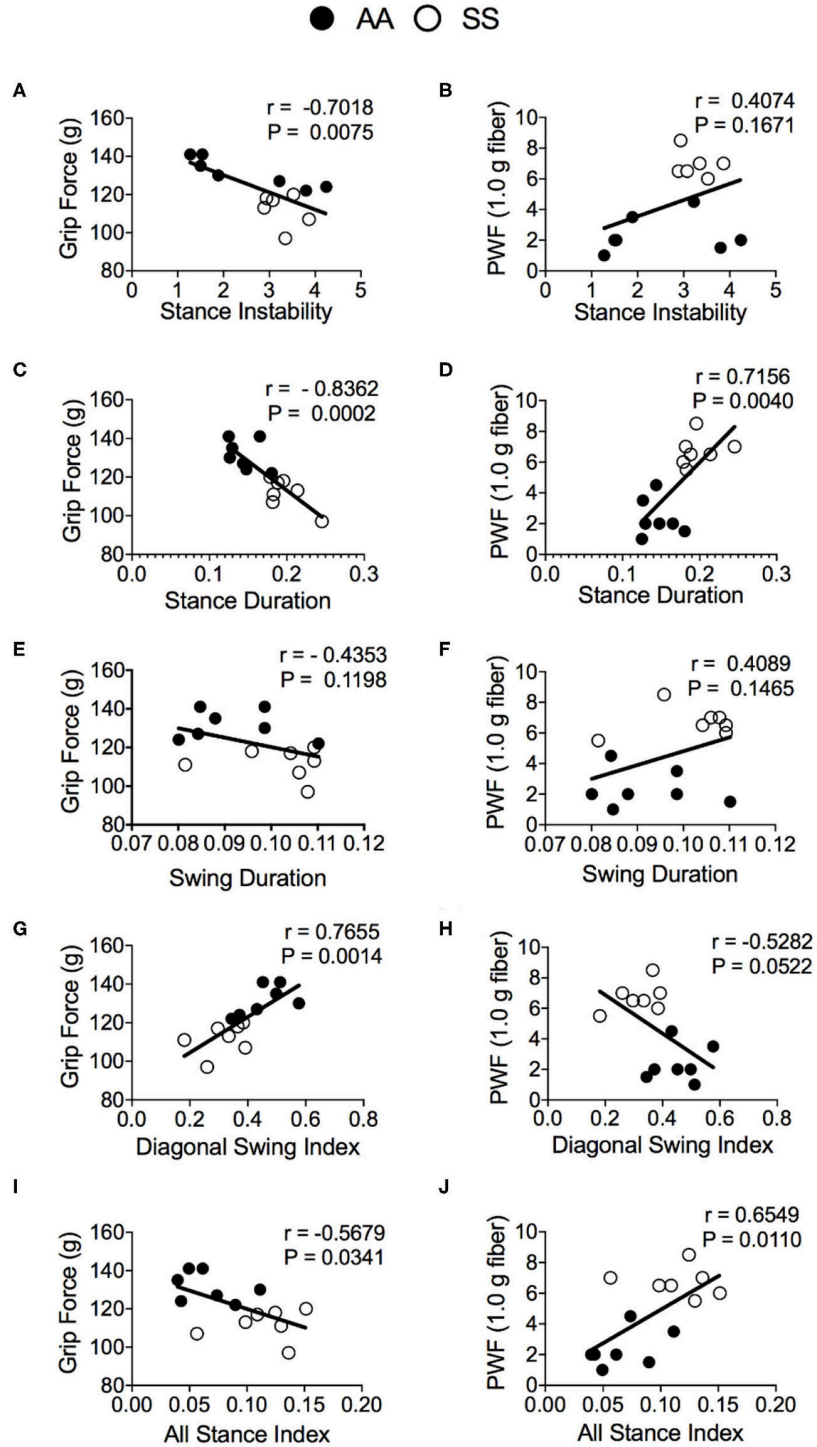
Correlation of gait parameters with deep tissue and mechanical hyperalgesia measures. Pearson correlation analysis was performed to detect associations of gait parameters with hyperalgesia in HbSS-BERK sickle (SS) and HbAA-BERK control (AA) mice. Correlation test results of grip force measures and paw withdrawal frequency (PWF) in response to 1.0-g von Frey filament, respectively, with stance instability **(A,B)**, stance duration **(C,D)**, swing duration **(E,F)**, diagonal swing index **(G,H)**, and all stance index **(I,J)** are shown. [*n* = 7 per group in **(A–J)**] A *p* < 0.05 was considered statistically significant. All data are represented as mean ± SEM.

To evaluate the utility of automated gait measurement as a complementary approach for assessing pain, we conducted a Pearson correlation analysis between conventional mechanical and deep tissue hyperalgesia measurements and gait parameters for combined groups of sickle and control mice. The stance instability demonstrated a strong correlation with deep tissue hyperalgesia (Figure 6A; *r* = −0.70, *p* = 0.0075) but no significant correlation with mechanical hyperalgesia (Figure 6B). However, the strongest correlation with hyperalgesia are observed for the stance duration with both grip force (Figure 6C; *r* = −0.84, *p* = 0.0002) and PWF (Figure 6D; *r* = 0.72, *p* = 0.004) indicating that stance duration is affected by existence of deep tissue and mechanical hyperalgesia. There was no correlation for the swing duration with both mechanical and deep tissue hyperalgesia (Figures 6E,F), even though there was a significant difference in swing duration between the sickle and control mice as described above. Among the swing indices, the diagonal swing index had strong (Figure 6G; *r* = 0.77, *p* = 0.0014) correlation with grip force and moderate (Figure 6H; *r* = −0.53, *p* = 0.0522) correlation with PWF, thus indicating a moderate decrease in the trot gait (walking with higher speed) with increasing hyperalgesia. All stance indices moderately correlated with both grip force (Figure 6I; *r* = −0.56, *p* = 0.034) and PWF (Figure 6J; *r* = 0.65, *p* = 0.011), demonstrating that anticipation of movement-evoked pain may contribute to more frequent hesitant stops with increasing pain.

## Discussion

Our results demonstrate increased caspase-3 activation in Purkinje cells of sickle mice compared to control mice, indicative of apoptosis and neurodegenerative changes in the cerebellar cortex. In sickle mice, the nucleolus is missing and the nucleus is smudged with condensed chromatin, clearly morphological signs of apoptosis. These misshapen nucleoli are immunopositive for cleaved caspase-3 (active form), a key protease in the apoptotic pathway and a known marker of cellular apoptosis including Purkinje cell apoptosis in the cerebellum (26–29). Therefore, increased cleaved caspase-3 positive immunostaining in the Purkinje cells of BERK sickle mice compared to control mice validates the histological observations of Purkinje cell apoptosis. It is likely that increased numbers of apoptotic Purkinje cells in the cerebellum of sickle mice contribute to alterations in their motor function leading to changes in gait. Gait characteristics during walking are significantly different in sickle mice compared to control mice perhaps due to increased Purkinje cell apoptosis. Purkinje cells provide primary outputs from the cerebellar cortex and are known to modulate motor functions.

Preclinical studies using transgenic sickle mice to elucidate mechanisms of sickle pain have evolved around traditional methods of stimulus-evoked hyperalgesia assessments (7) that are limited to the subjective nature of observations and an associated potential for bias (30). Most importantly, these methods performed in restraint and with noxious stimuli can generate stress (30). Therefore, objective measure of spontaneous pain without any stimuli are of growing interest. We have previously demonstrated that image analysis of facial expressions using mouse grimace scale (MGS) could detect pain in response to cold stimuli, although the method suffers from observer bias (31). Simultaneously, we tested the utility of static body length and curvature parameters (e.g., eccentricity of a fitted ellipse) extracted from image analysis as objective measures of hyperalgesia, and found that sickle mice had higher percent change in these parameters compared to control mice in response to cold stimuli, which was reversed upon analgesic treatment in sickle mice (31).

Complementary to the static gait changes sickle mice demonstrate alterations in walking gait parameters. Sickle mice displayed more stance instability and body sway from the center of the mass during their movement suggesting loss of balance during movement. Interestingly, human subjects with spinocerebellar ataxia type 6 (SCA6) standing on a flat surface demonstrate global stance instability with body sway that strongly correlates with disease severity (32). Human brain tissue examination and mouse models of SCA6 showed involvement of apoptotic Purkinje cells and their dysfunctional firing in SCA6–a predominantly hereditary neurodegenerative disease (33, 34). Therefore, higher stance instability in sickle mice suggests cerebellar Purkinje cell damage-associated disruption in sensorimotor processing of balance control. Sickle mice evinced significantly decreased walking speed, increased stance and swing duration, and also exhibit avoidance behaviors in hind paw gait parameters (increased hind paw bound swing), compared to control mice. Sickle mice consistently exhibited hesitation during recording for gait measurement, which is also reflected in their significantly increased swing and stance duration. Moreover, diagonal swing indices were significantly reduced in sickle mice in addition to reduced walking speed. Lower speed and increased reluctance to walk in sickle mice may be indicative of compensation for existing hyperalgesia and fear/anticipation of movement-evoked pain, respectively. Stance duration exhibited significantly positive correlation with deep tissue- and mechanical-hyperalgesia; and stance instability demonstrated significant correlation with deep tissue hyperalgesia. Also, diagonal swing indices and all stance indices demonstrate moderate to strong negative and positive correlation, respectively, with both deep tissue and mechanical hyperalgesia—indicating that gait compensation to avoid movement-evoked pain may contribute to reduced speed and increased hesitation during mobility. The evaluation of gait parameters, thus, can provide objective estimates of sickle pain devoid of observer bias and restraint-evoked stress in preclinical studies.

Alongside a large number of studies on experimental osteoarthritis (18), recent preclinical studies have tested the efficacy of automated gait analysis systems in murine models of neuropathic pain by spinal nerve injury (30, 35, 36), chronic constriction injury-induced pain [AU—(37)], CFA-induced pain (35, 36), paclitaxel-induced polyneuropathy (38) and post-operative pain in bone-reconstruction surgery (39). Commercially available systems such as CatWalk XT (Noldus) and GaitLab (ViewPoint Behavior Technology) rely on imaging paw prints reflected internally across an elevated glass floor where the animal walks (40). Other systems such as DigiGait (Mouse Specifics Inc.) and GaitScan/TreadScan (CleverSys) use video recordings to analyze paw prints of walking animals (41–43). However, these platforms do not offer user-customization according to specific needs as their software source codes are proprietary. On the contrary, *MouseWalker* system (used in this study) is a simpler system with walking apparatus assembled from readily available materials and inexpensive components, and the software is available free of cost (25).

Traditionally human sickle pain studies have relied on patient-reported visual analog scores or pain diaries, or relatively recent objective tools such as quantitative sensory testing (QST) (44–51). However, QST measures hypersensitivity to evoked thermal or mechanical stimuli. A 6-min walk study demonstrated that reduction of walking distance among children on hydroxyurea treatment and without cardiopulmonary complications correlated with history of silent strokes (52). Using 36-item short form (SF-36) survey that assesses quality of life (QoL) in patients, bodily pain that affects activities of daily life (ADL) was found to be significantly associated with chronic pain in thoracic spine and hip/lower limbs in adult sickle patients (53). In a study of children hospitalized for VOC, the rate of improvement of daily physical functions and rate of reduction in pain intensities were significant over the course of hospital stay, with negative effects in mood being associated proportionately with pain intensities and inversely with physical functioning scores (53). Therefore, negative scores of physical functioning or movement-gait changes may be indicative of pain. Peripheral neuropathy leading to acute loss of lower extremity mobility have been reported recently in sickle patients (54, 55). Our murine data indicates that if a similar correlation between pain scores and gait changes is seen in humans, monitoring gait in sickle patients to detect any onset/offset of abnormal or altered patterns may serve as tools to evaluate post-treatment improvement and/or monitoring chronic pain and associated QoL. Automated gait analysis using wearable technology has been used for objective detection of gradual improvement in physical functioning in post-operative period in patients with total hip arthroplasty (53). Thus, early assessment of dynamic gait features and locomotion deficits may help in early diagnosis of avascular necrosis and associated bone disorders prompting preventive measures.

An important characteristic of gait in sickle mice is the existence of longer and frequent hesitant stops reflected by significantly higher all stance indices compared to control mice which were also moderately associated with mechanical and deep tissue hyperalgesia independently–which may represent stalling due to fear or anticipation of movement-evoked pain. Autonomic nervous system (ANS) responses have been shown to be significantly disrupted in sickle patients (56). Vasoconstriction (or decrease in microvascular perfusion) is influenced greatly in response to anticipation of thermal pain in human sickle patients (57), and degree and rate of such neurally mediated-vasoconstriction is correlated with anxiety scores (51). While mental stress causes vasoconstriction in both sickle and healthy individuals (58), such vascular response may increase transit time of sickled RBCs and contribute to entrapment in microvasculature resulting in acute VOC. Additionally, neuroimaging analysis revealed that resting state functional connectivity is intensified in the locus coeruleus of the brain stems of SCD subjects compared to non-SCD anemic controls—indicating possibility of hyperactive sympathetic neurons contributing to modulation in peripheral microvascular blood flow (58). In patients with type1 and type2 diabetes (compared to healthy controls) decreased walking speed, more frequent stops and altered joint gait during movement were observed, while these patients also demonstrated 50% impairment in local tissue blood flow and other autonomic functions (vs. controls) (58). Thus, it is possible that gait alteration in sickle mice is both a function of existing pain and its effect on the sympathetic nervous system. Conversely, anticipation of pain may contribute to VOC. Thus, monitoring of gait characteristics of SCD subjects may provide information regarding prognosis of the disease, onset of acute crises and/or transition to chronic pain.

The role of cerebellar Purkinje cells in peripheral nociception is unclear. However, A-delta and C-fiber signals are relayed to Purkinje cells in the cerebellum (59–61) and nociceptive somatosensory and visceral signals stimulate Purkinje cell firing (62, 63). Interestingly, Purkinje cells in the vermis of the cerebellum project into the fastigial nucleus and these cerebellar structures are connected to cerebral areas controlling autonomic functions (64). Additionally, recent neuroimaging studies demonstrated enhanced cerebellar activity both in anticipation of and violation of expected level of pain (65, 66). Another study demonstrated overlapping cerebellar activity suggestive of pain-evoked motor adaptation (67). Therefore, Purkinje cell damage and altered stance behaviors in sickle mice with chronic pain in relation to sympathetic modulation of anticipated pain and pain-induced gait adaptation warrant further investigation.

## Conclusion

In conclusion, we provide first evaluation of walking-gait differences in sickle mice compared to control mice. Increased Purkinje cell apoptosis could contribute to altered movement leading to changes in gait. Importantly, several parameters of gait correlate with deep tissue and mechanical hyperalgesia in sickle mice. Thus, gait analysis can be used as a complementary and objective pain assessment tool devoid of stimuli-evoked techniques for assessing sickle pain. Recent advances in wearable technology offer the potential of monitoring gait from remote access in an unbiased and natural environment. Therefore, our observations provide a proof of principle to examine gait in SCD as a predictor of pain and other consequences of the disease.

## Data Availability Statement

The raw data supporting the conclusions of this article will be made available by the authors, without undue reservation.

## Ethics Statement

The animal study was reviewed and approved by Institutional Animal Care and Use Committee VA Long Beach Healthcare Center.

## Author Contributions

SK performed behavioral and histological analysis, wrote the manuscript, and prepared for submission. YW assembled the MouseWalker and performed MouseWalker experiments and collected the data. AA analyzed and interpreted the MouseWalker data, wrote the manuscript, and prepared for submission. DA performed behavioral analysis, preparation of tissues for histological analysis, and edited the manuscript. JL performed experiments and analyzed the data for hyperalgesia. VS analyzed and interpreted the data and prepared the figures. MT and SB extracted the parameters from video recordings for gait analysis. NL analyzed and interpreted pathology data, and prepared the pathology figures. KG developed the concept, designed, planned, and supervised the study, interpreted the data, and edited the manuscript. All authors contributed to the article and approved the submitted version.

## Conflict of Interest

KG reports grants from Grifols, 1910 Genetics and Cyclerion and honorarium from Novartis, Tautona Group, and CSL Behring, outside the submitted work. The remaining authors declare that the research was conducted in the absence of any commercial or financial relationships that could be construed as a potential conflict of interest.
